# Bacterial Iron Siderophore Drives Tumor Survival and Ferroptosis Resistance in a Biofilm‐Tumor Spheroid Coculture Model

**DOI:** 10.1002/advs.202404467

**Published:** 2024-08-12

**Authors:** Yoyo Wing Suet Yeung, Yeping Ma, Yanlin Deng, Bee Luan Khoo, Song Lin Chua

**Affiliations:** ^1^ Department of Applied Biology and Chemical Technology The Hong Kong Polytechnic University Kowloon Hong Kong SAR 999077 China; ^2^ Department of Biomedical Engineering City University of Hong Kong Hong Kong SAR 999077 China; ^3^ Hong Kong Center for Cerebro‐Cardiovascular Health Engineering (COCHE) Hong Kong SAR 999077 China; ^4^ City University of Hong Kong‐Shenzhen Futian Research Institute Shenzhen 518000 China; ^5^ State Key Laboratory of Chemical Biology and Drug Discovery The Hong Kong Polytechnic University Kowloon Hong Kong SAR 999077 China; ^6^ Research Centre of Deep Space Explorations (RCDSE) The Hong Kong Polytechnic University Kowloon Hong Kong SAR 999077 China

**Keywords:** biofilm, ferroptosis, *Pseudomonas aeruginosa*, pyoverdine, tumor microenvironment

## Abstract

Interactions between tumoral cells and tumor‐associated bacteria within the tumor microenvironment play a significant role in tumor survival and progression, potentially impacting cancer treatment outcomes. In lung cancer patients, the Gram‐negative pathogen *Pseudomonas aeruginosa* raises questions about its role in tumor survival. Here, a microfluidic‐based 3D‐human lung tumor spheroid‐*P. aeruginosa* model is developed to study the bacteria's impact on tumor survival. *P. aeruginosa* forms a tumor‐associated biofilm by producing Psl exopolysaccharide and secreting iron‐scavenging pyoverdine, which is critical for establishing a bacterial community in tumors. Consequently, pyoverdine promotes cancer progression by reducing susceptibility to iron‐induced death (ferroptosis), enhancing cell viability, and facilitating several cancer hallmarks, including epithelial–mesenchymal transition and metastasis. A promising combinatorial therapy approach using antimicrobial tobramycin, ferroptosis‐inducing thiostrepton, and anti‐cancer doxorubicin could eradicate biofilms and tumors. This work unveils a novel phenomenon of cross‐kingdom cooperation, where bacteria protect tumors from death, and it paves the way for future research in developing antibiofilm cancer therapies. Understanding these interactions offers potential new strategies for combatting cancer and enhancing treatment efficacy.

## Graphical abstract

A microfluidic‐based 3D‐lung tumor spheroid‐*Pseudomonas aeruginosa* model is developed to study tumor‐cell‐bacterial interactions. *P. aeruginosa* could form a tumor‐associated biofilm and secrete an iron‐scavenging pyoverdine. By removing excess iron from tumors, pyoverdine could reduce susceptibility of tumor cells to ferroptosis. A combinatorial treatment of ferroptosis‐inducing thiostrepton, antitumor doxorubicin, and tobramycin antibiotic could eradicate biofilms and tumors.

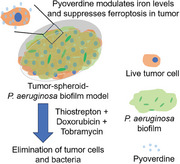

## 1 Introduction

Cancer remains a significant global health challenge, causing a substantial economic burden on healthcare systems worldwide. Cancer accounts for ten million deaths globally every year, where lung, colorectal, and breast cancer are the most common causes of death.^[^ ^1^ ^]^ The tumor microenvironment (TME) is a complex biological system, which plays a key role in cancer progression and treatment efficacy.^[^ ^2^ ^]^ Tumor cells can protect themselves from cell death pathways, such as apoptosis, necrosis, and autophagy. Recent studies showed that cancer cells exhibit dysregulated iron metabolism, making them highly susceptible to ferroptosis.^[^ ^3^ ^]^ Ferroptosis is a regulated form of cell death characterized by the accumulation of lipid peroxides, which leads to oxidative damage and, ultimately, cell demise.^[^ ^4^ ^]^ As a result, there has been growing interest in using ferroptosis induction as a potential anti‐cancer therapeutic approach against tumor cells.^[^ ^5^ ^]^ However, it is unclear how tumor cells circumvent ferroptosis for survival.

A neglected yet important component in the TME is bacteria associated with most tumors otherwise thought to be sterile.^[^ ^6^ ^]^ Tumor‐associated bacteria, such as *Pseudomonas* and *Staphylococcus* species,^[^ ^7^ ^]^ are highly prevalent in lung cancer, especially in the elderly (85%). The role of tumor‐associated bacteria in tumors is complex and multi‐faceted, such as *Fusobacterium nucleatum*, which can increase tumor growth and metastasis of breast cancer via modulation of the immune system.^[^ ^8^ ^]^ Moreover, tumor‐associated bacteria form multicellular communities termed biofilms and colonize tumors such as oral squamous cell carcinoma and gallbladder cancer.^[^ ^9^ ^]^

Understanding the precise mechanisms by which tumor‐associated bacteria influence tumor growth via interactions with cancer cells and anti‐cancer treatment response is important. Few models can study tumor‐bacterial interactions to obtain 3D cocultures and failed to reproduce the dynamics of cells in vivo accurately. This raises the need to develop convenient, high‐throughput in vitro models that accurately mimic tumor spheroid‐bacterial interactions and TME. We recently developed a clinically relevant 3D microfluidic‐based tumor spheroid‐bacteria model.^[^ ^10^ ^]^ that supported that tumor‐associated bacteria could establish biofilm on tumor cells and promote tumor progression in bladder cancer.

To address how tumor‐associated bacteria could protect tumor cells from ferroptosis, we developed a novel lung tumor spheroid model incorporating cancer cells and bacterial biofilms to evaluate the effect of biofilms on tumor microenvironment and survival. We showed for the first time that a bacterial biofilm‐secreted iron siderophore, pyoverdine, could protect tumor cells from ferroptosis‐induced death via iron chelation, leading to tumor survival and epithelial‐to‐mesenchymal transition in tumor progression. Mutant biofilms without pyoverdine‐synthesizing ability could not prevent tumor death by ferroptosis. Antibacterial antibiotics, ferroptosis‐inducing thiostrepton, and antitumor doxorubicin could eliminate tumor cells and tumor‐associated bacteria. Our work revealed the potential mechanism of biofilms in protecting tumor cells from ferroptosis and provided fresh insights into antibiofilm‐antitumor combinatorial therapy for improved cancer chemotherapy.

## 2 Results

### 2.1 Establishment of a Microfluidic Model

**Figure 1:**
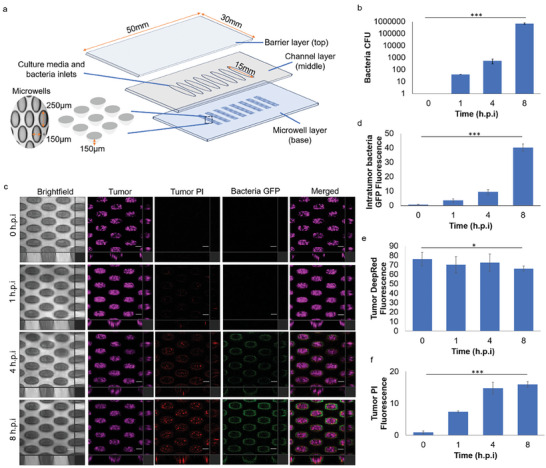
Establishment of 3D‐human lung tumor spheroid‐*P. aeruginosa* interactions model. a) Schematic diagram of microfluidics chip for culturing 3D‐lung tumor spheroid‐tumor‐associated *P. aeruginosa* coculture model. b) Population number of intratumor *P. aeruginosa* in lung tumor spheroid over time. c) Representative images of 3D‐lung tumor spheroid‐tumor‐associated *P. aeruginosa* coculture model over time. Scale bar: 100 µm. d) Relative GFP fluorescence levels of intra‐tumor *P. aeruginosa* in lung tumor spheroid over time. e) Relative Deep‐Red fluorescence levels of lung tumor spheroids in tumor‐bacterial coculture model over time. f) Relative PI fluorescence levels of lung tumor spheroids in tumor‐bacterial coculture model over time. The means and s.d. from triplicate experiments from 3 independent trials were shown. ***p < 0.001.

To reflect the tumor spheroid‐biofilm interactions in a dynamic TME in vivo and study the effects of biofilm‐secreted metabolites on cancer progression under well‐defined conditions, we developed a novel microfluidic model incorporating 3D‐lung tumor spheroids and tumor associated *Pseudomonas aeruginosa* biofilms (**Figure** **1**a, Figure S1a, Supporting Information), which comprises of the three components:^[^ ^1^ ^]^ top layer with inlets for the introduction of cells;^[^ ^2^ ^]^ an intermediate layer for fluid movement and retention.^[^ ^3^ ^]^ bottom microwell layer for tumor cluster formation. The microwell mold was fabricated by photolithography, and the device was fabricated by polydimethylsiloxane (PDMS). The 70% ethanol‐sterilized device was coated in 2.5% bovine serum albumin, and each microchannel was seeded with 5 × 10^4^ cells mL^−1^ A549 lung tumor cells to establish the 3D coculture culture. The resulting tumor size range (Figure S1b, Supporting Information) would be clinically comparable to large tumor microclusters in vivo (≈50 cells).^[^ ^10^ ^]^

To mimic the different magnitudes of tumor associated bacteria within the TME, we first established the tumor spheroid‐ bacterial coculture model at various initial multiplicity of infection (MOI) rates (human cells: bacteria). We showed that increased bacterial infection (Figure S2a–c, Supporting Information) was correlated with the rapid death and collapse of tumors (Figure S2d,e, Supporting Information). The initial MOI that enabled the co‐survival of tumor and tumor‐associated bacteria was 10^5^:1.

Based on prior studies showing the infection period for rapid establishment of the coculture model, we selected 1, 4, and 8 h post‐infection (h.p.i) as various time points to evaluate the coculture model over time. We quantified the number of bacteria within the lung tumor spheroids using the CFU assay (Figure 1b), so there were an estimated of 10^6^ bacterial cells mL^−1^ (around 3 × 10^3^ bacteria per microwell) by 8 h.p.i. We observed the localization of tumor associated bacteria on the lung tumor spheroids using confocal laser scanning microscopy (CLSM) (Figure 1c–f), indicating that *P. aeruginosa* could colonize the lung tumors by 8 h.p.i. Hence, we chose 8 h.p.i for subsequent experiments in our study, *P. aeruginosa* numbers eventually reached 10^6^ cells mL^−1^ by 8 h.p.i, corresponding to the ratios previously validated in animal cancer models and patient tumor sections.^[^ ^11^ ^]^ It is also important to note that media was consistently replaced every 2 h to prevent nutrient depletion, and its associated deaths of bacteria and tumor cells.

To evaluate if apoptosis could play a role in tumor cell death, we employed Annexin V dye for apoptosis^[^ ^12^ ^]^ and found minimal apoptotic tumor cells within the entire experimental duration (Figure S3, Supporting Information). This indicated that apoptosis was not involved in the killing of tumor cells.

### 2.2 Biofilm Formation Is Important for Establishing Tumor‐Associated Bacterial Communities

**Figure 2:**
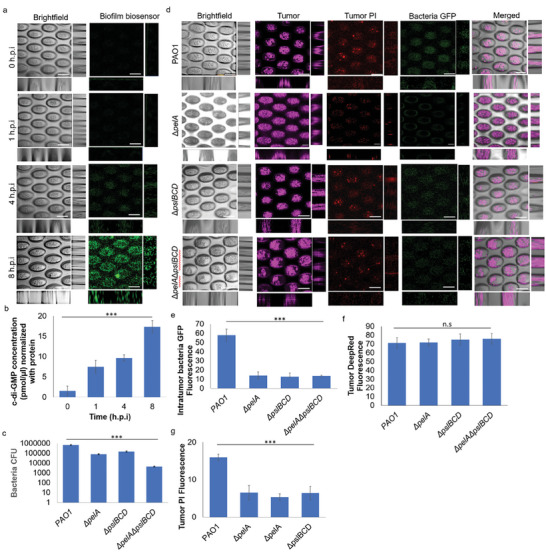
Biofilms are important for bacterial colonization in tumors. a) Representative images of 3D‐lung tumor spheroid‐tumor‐associated *P. aeruginosa* PAO1/p*_cdrA_*‐*gfp* coculture model over time. Scale bar: 200 µm. b) Relative intracellular c‐di‐GMP levels of tumor‐associated *P. aeruginosa* in the tumor‐bacterial co‐culture model, as quantified by ELISA. c) CFU assay of intratumor wild‐type PAO1 and its exopolysaccharide mutants in lung tumor spheroid showed exopolysaccharides were important for bacterial colonization within tumors. d) Representative images comparing wild‐type PAO1 and exopolysaccharide‐deficient mutants in the tumor‐bacterial co‐culture model. Scale bar: 200 µm. e) Relative GFP fluorescence levels of intratumor *P. aeruginosa* PAO1 and its mutants in the tumor‐bacterial coculture model. f) Relative deep‐red fluorescence levels of lung tumor spheroids in a tumor‐bacterial coculture model. g) Relative PI fluorescence levels of lung tumor spheroids in a tumor‐bacterial coculture model. The means and s.d. from triplicate experiments from 3 independent trials were shown. ***p < 0.001.

Since we observed that tumor‐associated bacteria were clustered together in a community (Figure 1c), we next asked if they actively participated by forming biofilms or were just passively present in the tumor spheroids. We employed two well‐established biofilm biomarkers, p*_cdrA_*‐*gfp* and c‐di‐GMP, whose active expressions were correlated to biofilm formation,^[^ ^13^ ^]^ to evaluate tumor‐associated bacteria in the co‐culture model. The CdrA is an adhesion protein key to binding *P. aeruginosa* to sticky exopolymeric matrix,^[^ ^14^ ^]^ while c‐di‐GMP is a universal secondary messenger that regulates biofilm formation.^[^ ^15^ ^]^ We observed that tumor‐associated *P. aeruginosa* upregulated the biofilm biosensor by 8 h.p.i (**Figure** **2**a), which corroborated with the ELISA quantification of c‐di‐GMP within *P. aeruginosa* cells (Figure 2b).

Our prior studies had shown that biofilms formed on tumors, but the biofilm matrix component important in establishing biofilms on the tumor remains unclear.^[^ ^10^ ^]^ We next asked which biofilm matrix component was important in establishing tumor‐associated *P. aeruginosa* biofilms. We screened our in‐house bacterial mutant library deficient in biofilm matrix synthesis (Figure S4, Supporting Information). We showed that Δ*pelA*Δ*pslBCD* deficient in Pel and Psl exopolysaccharide production could not establish effective biofilms on the tumor spheroids (Figure 2c–g). This indicated both exopolysaccharides were critical for biofilm formation by tumor‐associated *P. aeruginosa*.

We also evaluated if *P. aeruginosa* could be accumulated intracellularly within the tumor cells, by washing away the biofilm bacteria and quantifying the intracellular bacteria population. We observed minimal increase in intracellular bacterial numbers over time (Figure S5a, Supporting Information), indicating that there was minimal intracellular accumulation of bacteria within host tumor cells. There was also minimal ROS in the tumor cells that was associated to intracellular bacteria (Figure S5b, Supporting Information). This showed intracellular bacteria did not have significant effects on the tumor cells as compared to biofilm bacteria.

### 2.3 *Pseudomonas aeruginosa* Protects Tumor Spheroids from Ferroptosis

**Figure 3:**
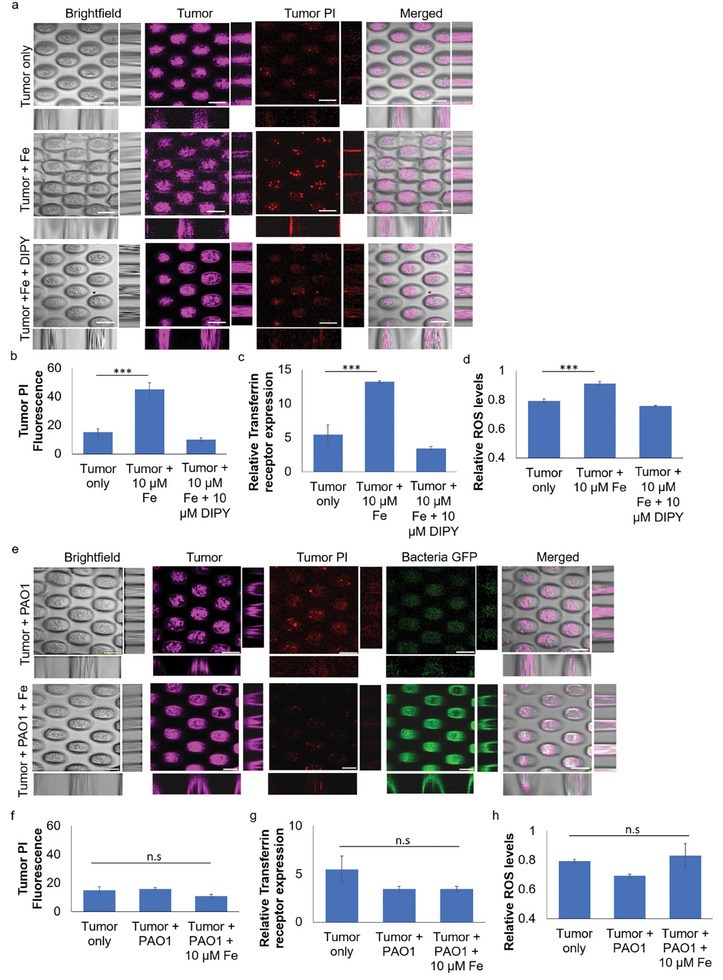
*P. aeruginosa* is important in the suppression of ferroptosis in tumor cells. a) Representative images of tumors treated with exogenous iron or DIPY, where exogenous iron addition could cause the death of tumor, while exogenous DIPY treatment could reduce the death of tumor cells. Scale bar: 200 µm. b) Relative PI fluorescence levels of lung tumor spheroids after exogenous iron or DIPY treatment. c) Relative transferrin receptor expression by tumor cells after exogenous iron or DIPY treatment. d) Relative ROS levels within tumor cells after exogenous iron or DIPY treatment. e) Representative images of 3D‐lung tumor spheroid‐tumor‐associated *P. aeruginosa* coculture model after exogenous iron treatment. Scale bar: 200 µm. f) Relative PI fluorescence levels of lung tumors in the tumor‐bacterial coculture model after exogenous iron treatment. g) Relative transferrin receptor expression by lung tumors in the tumor‐bacterial co‐culture model after exogenous iron treatment. h) Relative ROS levels within lung tumor cells in the tumor‐bacterial co‐culture model after exogenous iron treatment. The means and s.d. from triplicate experiments from 3 independent trials were shown. ***p < 0.001.

Ferroptosis plays a significant role in tumor suppression, and an increase in iron uptake can enhance tumor cell sensitivity to ferroptosis.^[^ ^16^ ^]^ Hence, we altered the iron levels, by either increasing or removing iron ions within the coculture to observe the effects on ferroptosis. We showed that the exogenous addition of iron to tumor spheroids increased the killing of tumor spheroids. In contrast, the addition of iron chelator 2,2′‐dipyridyl (DIPY) mitigated the killing of iron‐treated tumor spheroids (**Figure** **3**a,b), which corroborated with findings from previous studies.^[^ ^17^ ^]^

To evaluate if the tumor spheroids were undergoing ferroptosis, we evaluated the expression of the transferrin receptor, previously established as a specific ferroptosis biomarker,^[^ ^18^ ^]^ and intracellular ROS levels, which were highly produced during ferroptosis.^[^ ^19^ ^]^ The iron‐treated tumor spheroids possessed high expression of transferrin receptors (Figure 3c). They heightened ROS levels (Figure 3d) than nontreated cells, indicating that ferroptosis occurred in our model.

Surprisingly, the presence of tumor‐associated *P. aeruginosa* biofilms could prevent the death of iron‐treated tumor cells (Figure 3e,f), where they possessed reduced expression of transferrin receptor (Figure 3g) and intracellular ROS levels (Figure 3h), showing that ferroptosis in tumor cells was suppressed by tumor‐associated *P. aeruginosa*. Our findings apply to different cancer cell lines (Figure S6, Supporting Information), indicating that *P. aeruginosa* was involved in ferroptosis suppression in tumor TME.

### 2.4 *P. aeruginosa* Pyoverdine Protects Tumors from Ferroptosis

To study which component from the tumor‐associated bacterial biofilms contributes to ferroptosis suppression by tumor cells, we first showed that biofilm supernatant could prevent the death of tumor cells in a similar manner and prolonged heating to denature proteins did not eliminate the ferroptosis suppression activity (Figure S7, Supporting Information). This indicated a secreted non‐protein metabolite from the biofilms involved in ferroptosis suppressing the tumor spheroids.

**Figure 4:**
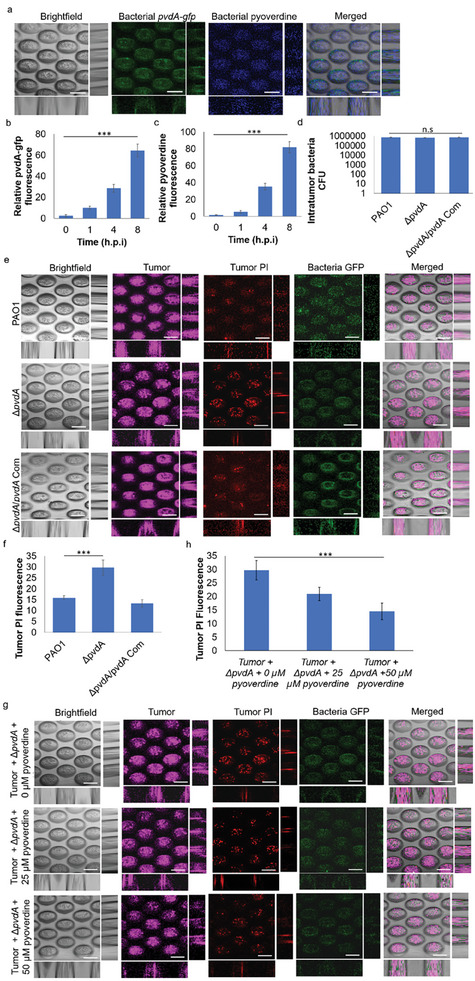
*P. aeruginosa p*yoverdine is important in suppressing ferroptosis‐led death in tumor cells. a) Representative images of 3D‐lung tumor spheroid‐tumor‐associated *P. aeruginosa* PAO1/p*_pvdA_*‐*gfp* coculture model. Scale bar: 200 µm. b) Relative GFP fluorescence levels of PAO1/p*_pvdA_*‐*gfp* in a tumor‐bacterial coculture model. c) Relative pyoverdine fluorescence levels of PAO1/p*_pvdA_*‐*gfp* in a tumor‐bacterial coculture model. d) CFU assay of intra‐tumor wild‐type PAO1 and its pyoverdine mutant in lung tumor spheroid showed pyoverdine was not important for bacterial colonization within tumors. e) Representative images of 3D‐lung tumor spheroid‐tumor‐associated *P. aeruginosa* PAO1 and its pyoverdine mutants, all tagged with p*_lac_*‐*gfp*. Scale bar: 200 µm. f) Relative PI fluorescence levels of lung tumors cultured with *P. aeruginosa* PAO1 and its pyoverdine mutants in the tumor‐bacterial coculture model. g) Representative images of 3D‐lung tumor spheroid‐tumor‐associated pyoverdine mutant bacteria treated with exogenous pyoverdine addition. Scale bar: 200 µm. h) Relative PI fluorescence levels of lung tumors cultured with pyoverdine mutant bacteria treated with exogenous pyoverdine addition in the tumor‐bacterial coculture model. The means and s.d. from triplicate experiments from 3 independent trials were shown. ***p < 0.001. n.s: not significant.

Since *P. aeruginosa* biofilms secrete pyoverdine, an iron siderophore for scavenging iron (both Fe^2+^ and Fe^3+^) from the environment,^[^ ^20^ ^]^ we hypothesized that pyoverdine was involved in ferroptosis suppression in tumor cells. We showed using CLSM that tumor‐associated *P. aeruginosa* upregulated the expression of pyoverdine synthesis gene *pvdA* and pyoverdine over time (**Figure** **4**a–c). While the ability to synthesize pyoverdine was not highly important for the colonization of *P. aeruginosa* in tumors (Figure 4d), pyoverdine was important for the survival of tumor cells. Loss of ability to synthesize pyoverdine by Δ*pvdA* abolishes tumor cells' survival ability. In contrast, restoration of pyoverdine synthesis in the complementation strain Δ*pvdA*/*pvdA* Com could recover the bacterial ability to protect the tumor cells (Figure 4e,f). To mitigate the effects caused by loss of pyoverdine, we also employed the specific ferroptosis inhibitor, ferrostatin, which could also prevent ferroptosis of Δ*pvdA* mutant‐treated tumor spheroids, where lower levels of ROS and expression of transferrin receptor were observed (Figure S8, Supporting Information).

Our findings have broader implications beyond the specific cancer cell line studied. Using other cancer cell lines, we observed that pyoverdine‐deficient bacteria could not shield the tumor cells from ferroptosis, as demonstrated in Figure S6 (Supporting Information). This indicates that the interactions between tumor‐associated bacteria and tumor cells, mediated by pyoverdine, might play a crucial role in evading ferroptosis in various cancer types. Next, we showed that the exogenous addition of pyoverdine to tumor cells with tumor‐associated Δ*pvdA* mutant could also serve similar purposes as the original bacteria in protecting the tumor cells from ferroptosis, where pyoverdine could ensure the survival of the tumor cells (Figure 4g,h). This indicated that pyoverdine is important in ferroptosis suppression in tumor cells.

### 2.5 Pyoverdine Modulates Iron Levels in Tumor Spheroids

**Figure 5:**
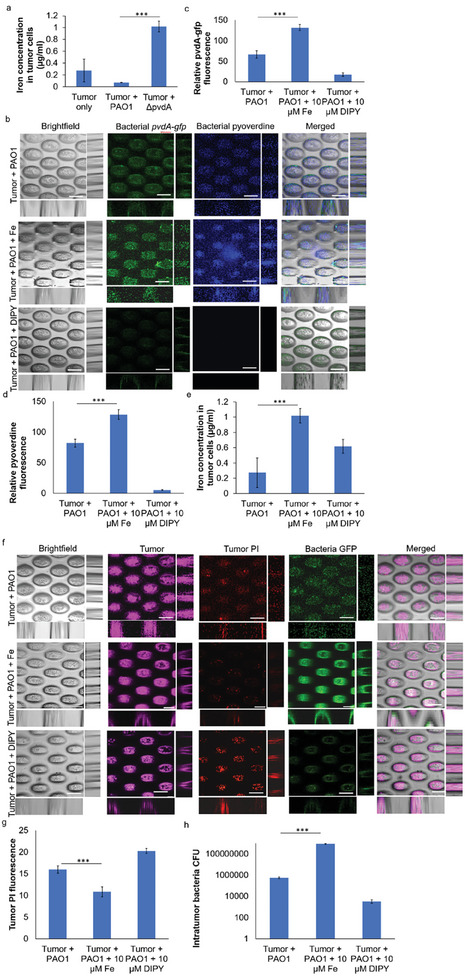
*P. aeruginosa* pyoverdine modulates iron levels in tumor spheroids. a) Intracellular iron concentration in tumor spheroids containing tumor‐associated *P. aeruginosa* PAO1 and its pyoverdine mutant. b) Representative images of 3D‐lung tumor spheroid‐tumor‐associated *P. aeruginosa* PAO1/p*_pvdA_*‐*gfp* coculture model treated with exogenous iron or DIPY. Scale bar: 100 µm. c) Relative GFP fluorescence levels of PAO1/p*_pvdA_*‐*gfp* in tumor‐bacterial co‐culture model treated with exogenous iron or DIPY. d) Relative pyoverdine fluorescence levels of PAO1/p*_pvdA_*‐*gfp* in tumor‐bacterial coculture model treated with exogenous iron or DIPY. e) Intracellular iron concentration in tumor spheroids containing tumor‐associated *P. aeruginosa* PAO1 treated with exogenous iron or DIPY. f) Representative images of 3D‐lung tumor spheroid‐tumor‐associated *P. aeruginosa* PAO1/p*_lac_*‐*gfp* coculture model treated with exogenous iron or DIPY. Scale bar: 100 µm. g) Relative PI fluorescence levels of lung tumors cultured with tumor‐associated *P. aeruginosa* PAO1 in the tumor‐bacterial coculture model. h) CFU assay of intra‐tumor wild‐type PAO1 in lung tumor spheroids treated with exogenous iron or DIPY. The means and s.d. from triplicate experiments from 3 independent trials were shown. ***p < 0.001.

Pyoverdine is a metal scavenger with a high affinity for iron, so we next evaluate how pyoverdine alters iron levels in tumor spheroids to achieve ferroptosis suppression. We quantified the iron concentrations in the tumor cells, where tumor cells possessing wild‐type *P. aeruginosa* had significantly lower iron levels than those with ΔpvdA mutant (**Figure** **5**a), suggesting that lower iron levels correlated to ferroptosis suppression. Pyoverdine could also enter into the tumor cells with minimal cytotoxicity (Figure S9, Supporting Information), which corroborated with other studies that pyoverdine could accumulate in human cells with low toxicity.^[^ ^21^ ^]^

Next, we also showed that tumor‐associated *P. aeruginosa* could also help to modulate changes in the iron concentration within tumor spheroids. Exogenous addition of FeCl_3_ enhanced the pvdA gene's expression and stimulated pyoverdine production by tumor‐associated wild‐type *P. aeruginosa* (Figure 5b–d). Conversely, the opposite effect was observed when DIPY was added (Figure 5b–d). The iron levels remained largely stable within the tumor cells in the presence of tumor‐associated *P. aeruginosa*, even with FeCl_3_ or DIPY treatment (Figure 5e). Hence, the modulation of stable iron levels ensured a consistent death rate of tumor cells even in the presence of FeCl_3_ or DIPY treatment (Figure 5f,g). On the other hand, changes in iron levels affect the survival of *P. aeruginosa* instead, where we observed higher and lower bacterial numbers with FeCl_3_ treatment and DIPY treatment, respectively (Figure 5h).

### 2.6 Pyoverdine Enables Tumor Epithelial‐to‐Mesenchymal Transition (EMT) Progression

Tumor progression is marked by the heightened invasiveness of tumors, which is closely associated with their increased metastatic potential.^[^ ^22^ ^]^ Here, we aim to evaluate the expression of EMT biomarkers (CD44, CD133 and CD166) in the tumor cells after colonization by tumor‐associated bacteria. CD44 is a surface glycoprotein important in tumor initiation, and metastasis is used as a cancer stem cell biomarker.^[^ ^23^ ^]^ CD133 is a cancer stem cell (CSC) biomarker in lung tumors,^[^ ^24^ ^]^ while CD166 promotes cancer stem‐likeness in primary cancer cells.^[^ ^25^ ^]^

**Figure 6:**
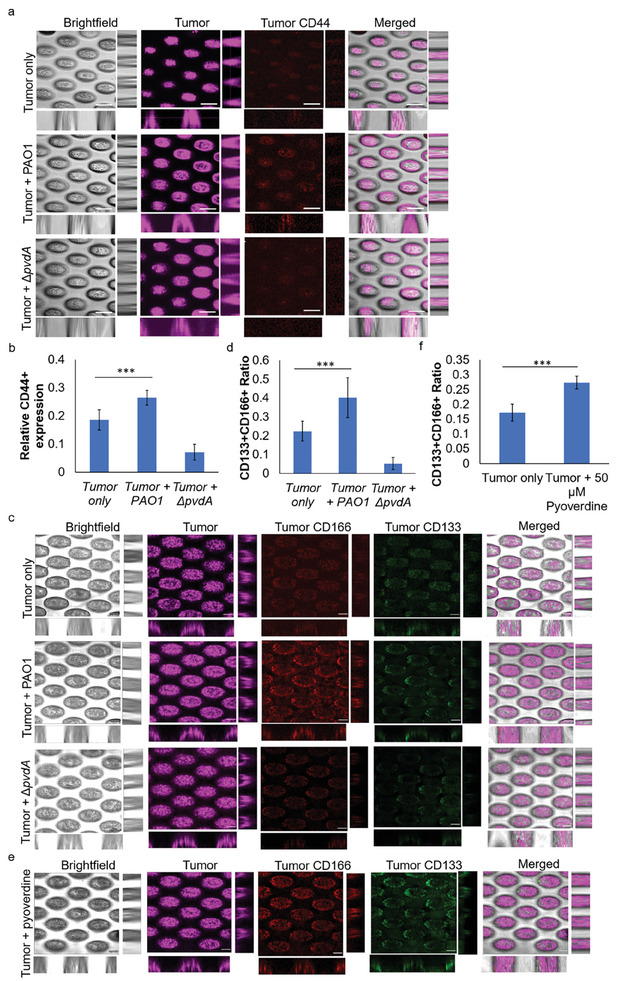
Pyoverdine enables tumor epithelial‐to‐mesenchymal transition (EMT) progression. a) Representative images of CD44 expression by 3D‐lung tumor spheroid cultivated with tumor‐associated *P. aeruginosa* PAO1 or pyoverdine mutant. Scale bar: 100 µm. b) Relative CD44 expression by tumor cells cultivated with tumor‐associated *P. aeruginosa* PAO1 or pyoverdine mutant. c) Representative images of CD133 and CD166 expression by 3D‐lung tumor spheroid cultivated with tumor‐associated *P. aeruginosa* PAO1 or pyoverdine mutant. Scale bar: 100 µm. d) CD133^+^/CD166^+^ ratio of tumor cells cultivated with tumor‐associated *P. aeruginosa* PAO1 or pyoverdine mutant. e) Representative images of CD133 and CD166 expression by 3D‐lung tumor spheroid treated only with exogenous pyoverdine. Scale bar: 100 µm. f) CD133^+^/CD166^+^ ratio of tumor cells treated only with exogenous pyoverdine. The means and s.d. from triplicate experiments from 3 independent trials were shown. ***p < 0.001.

We showed that tumors with tumor‐associated wild‐type *P. aeruginosa* had increased CD44 biomarker expression compared to tumor cells with tumor‐associated Δ*pvdA* mutant (**Figure** **6**a,b). At the same time, tumor spheroids upregulated CD133 and CD166 upon co‐culturing with tumor‐associated wild‐type *P. aeruginosa*, indicating the expression of cancer stem cells (Figure 6c,d).

Similarly, the direct addition of exogenous pyoverdine to tumor‐associated bacteria‐absent tumor spheroids could also improve cancer cell survival and drive the EMT transition in the tumor spheroids (Figure 6e,f). This indicated that bacterial secreted metabolite within the TME can enable the tumor progression.

### 2.7 Thiostrepton+Antibiotic (Tobramycin) +Anticancer (Doxorubicin) Combinatorial Treatment Eliminates Tumor‐Bacterial Coculture

**Figure 7:**
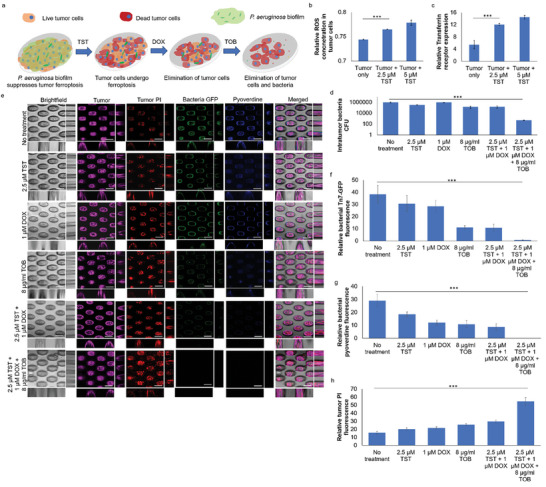
Triple treatment of thiostrepton (TST), tobramycin (TOB) and doxorubicin (DOX) can eliminate tumor‐bacterial coculture. a) Scheme of TST‐TOB‐DOX triple treatment on tumor and tumor‐associated *P. aeruginosa*. TST could induce b) ROS and c) transferrin receptor expression in tumors. d) CFU assay of intratumor wild‐type PAO1 in lung tumor spheroids treated with TST‐TOB‐DOX monotherapies or combinatorial therapy. e) Representative images of 3D‐lung tumor spheroid‐tumor‐associated *P. aeruginosa* co‐culture model treated with TST‐TOB‐DOX monotherapies or combinatorial therapy. Scale bar: 100 µm. f) Relative GFP fluorescence levels of tumor‐associated *P. aeruginosa* PAO1 in the tumor‐bacterial coculture model after TST‐TOB‐DOX monotherapies or combinatorial therapy. g) Relative pyoverdine fluorescence levels of tumor‐associated *P. aeruginosa* PAO1 in the tumor‐bacterial coculture model after TST‐TOB‐DOX monotherapies or combinatorial therapy. h) Relative PI fluorescence levels of lung tumors cultured with tumor‐associated *P. aeruginosa* PAO1 in the tumor‐bacterial coculture model after TST‐TOB‐DOX monotherapies or combinatorial therapy. The means and s.d. from triplicate experiments from 3 independent trials were shown. ***p < 0.001.

Lastly, we aim to develop a novel strategy to eliminate tumor and tumor‐associated bacteria with a combination of a pro‐ferroptosis inducer, an antimicrobial and an anti‐cancer drug (**Figure** **7**a scheme). We chose thiostrepton as a pro‐ferroptosis inducer because of its ability to induce ferroptosis.^[^ ^26^ ^]^ and more interestingly, it could also act as pyoverdine uptake inhibitor,^[^ ^27^ ^]^ indicating that thiostrepton may impose dual effects on both the tumor cells and tumor‐associated bacteria. To address the efficacy of thiostrepton, we showed that thiostrepton could induce ROS (Figure 7b) and activate the expression of the transferrin receptor (Figure 7c) in the tumor‐associated bacteria colonized tumors. From the point of view of *P. aeruginosa*, we also confirmed that the thiostrepton concentration used in this study could inhibit *pvdA* expression and pyoverdine production without killing *P. aeruginosa* pure culture (Figure S10, Supporting Information).

Although thiostrepton could impose inhibitory effects on both tumor‐associated *P. aeruginosa* and tumor cells, it was insufficient to eliminate them completely. This raises the rationale to introduce an anti‐cancer agent and antibiotic to kill tumor cells and tumor‐associated *P. aeruginosa* respectively. We chose tobramycin as the antibiotic and doxorubicin as the anti‐cancer agent^[^ ^10^ ^]^ as a proof‐of‐concept to validate our triple drug combinatorial therapy. The triple therapy was most effective in eliminating *P. aeruginosa*, as observed in CFU assay and CLSM (Figure 7d–f) and inhibiting pyoverdine production (Figure 7g), compared to monotherapy and no‐treatment control. Furthermore, the triple therapy could significantly reduce the viability of tumors (Figure 7h), confirming that the thiostrepton and doxorubicin could eliminate the tumor cells effectively. Hence, the triple combinatorial therapy could eliminate tumor and tumor‐associated bacteria in the coculture model.

To further address the role of immune system in the combinatorial treatment, we introduced macrophages to show that macrophages could associate with the tumor cells and bacterial biofilms (Figure S11a, Supporting Information). With the addition of combinatorial treatment, there was significant elimination of tumor spheroids (Figure S11b, Supporting Information). We also observed increased production of inflammatory cytokines (TNF‐α, IL‐2 and IL‐6) by the macrophages (Figure S11c–e, Supporting Information), suggesting that the combinatorial treatment could improve activation of immune response.

## 3 Discussion

Most cancer‐bacterial interaction studies use 2D cultures or hydrogels to establish 3D cultures, but such methods typically require long incubation durations with low throughput and clinical relevance. Using in vivo animal models is also highly challenging and expensive, which warrants developing a clinically relevant 3D model. Hence, applying novel microfluidic‐based technologies for evaluating novel tumor‐associated bacterial interactions, drug testing and tumor monitoring under well‐defined settings is important. Moreover, these studies were mainly focused on using microbes as an anti‐tumor therapy,^[^ ^28^ ^]^ whereas our work was distinct in studying how resident intratumor bacteria, notably *P. aeruginosa*, can benefit tumor survival.

The presence of tumor‐associated bacteria in the TME adversely affects cancer treatment. Tumor‐associated bacteria may interfere with the immune clearance of tumors or induce mutations in cancer cells. Moreover, *P. aeruginosa* is inherently resistant to numerous antibiotics, and its presence within tumor tissues may contribute to antibiotic resistance development. This resistance can hinder effective cancer therapy, making infections harder to treat and potentially compromising patient outcomes.

Since microbes can release many metabolites into the environment, their released metabolites could likely alter TME and tumor behavior. Although iron availability is often limited in many environments and iron competition between bacteria and host is common in infections,^[^ ^29^, ^30^ ^]^ we showed that tumor‐associated bacteria provided cross‐kingdom assistance to the tumor cells by suppressing ferroptosis by producing iron scavengers. Pyoverdine acts as a high‐affinity iron chelator, tightly binding to iron ions and facilitating their uptake into *P. aeruginosa* cells. This creates a “win‐win” situation for the bacterium, as it benefits from iron recovery, promoting the growth of its biofilm. By efficiently scavenging iron from its surroundings, *P. aeruginosa* gains a survival advantage, enhancing its ability to thrive in the environment. This is a surprising finding as *P. aeruginosa* is highly cytotoxic against host cells by producing different virulence factors, such as pyocyanin and exotoxin A.^[^ ^31^ ^]^ Since biofilms are typically less cytotoxic than planktonic bacteria,^[^ ^32^ ^]^ it could be possible that tumor‐associated *P. aeruginosa* biofilms were less cytotoxic and could co‐exist with tumor cells.

On the other hand, tumor cells gain resistance to ferroptosis despite having known to possess dysfunctional iron metabolism and resultant susceptibility to ferroptosis inducers. Ferroptosis is associated with resistance to cancer therapy,^[^ ^33^ ^]^ so ferroptosis suppression in tumor cells can enable the survival of tumors. Moreover, tumor progression is a multifactorial process, which growth factors and cross‐talk between tumor cells and surrounding stroma cells or inflammatory cells can be promoted. We provided alternative insight that tumor‐associated bacteria can also enable tumor progression via ferroptosis suppression via the production of pyoverdine.

Improving cancer therapy raises the need to consider tumor‐associated bacteria's role in ferroptosis suppression and develop novel strategies to eliminate tumor‐associated bacteria.^[^ ^34^ ^]^ We proposed a novel triple‐treatment strategy to eliminate tumor cells and tumor‐associated bacteria. Thiostrepton has dual effects on both tumor cells and tumor‐associated bacteria, where it induces ferroptosis of tumor cells and inhibits pyoverdine production by *P. aeruginosa*. Hence, the thiostrepton‐antibiotic‐anti‐cancer drug combinatorial therapy could successfully eliminate tumors and associated tumor‐associated bacteria.

The TME is much more complex in clinical settings, especially when microbes play an active role in tumor survival and progression. Bacterial biofilms may block effective anti‐cancer treatment and interfere with proper immune function.^[^ ^10b^ ^]^ Furthermore, biofilms can disperse,^[^ ^35^ ^]^ potentially facilitating the dissemination of tumor cells and promoting metastasis. Considering these factors, we envision that our coculture model can be effectively applied using patient‐derived tumor models under precisely controlled conditions in clinical settings. This approach can offer valuable insights for mechanistic studies and aid in discovering novel drugs for cancer treatment. In conclusion, we provide new insights into the TME involving cross‐kingdom interactions of tumors and tumor‐associated bacteria and offer potential antimicrobial‐anticancer treatment for effective tumor therapy.

## 4 Experimental Section

### Design and Fabrication of Microfluidics Chip

The microfluidics chip was designed with 300 microwells per array with a depth of 6 mm, where each tapered microwell had a size of 150 × 250 × 150 µm (length × width × depth). Polydimethylsiloxane (PDMS) was used as the material for the microfluidics chip. It was prepared by mixing the base resin and curing agent at a 10:1 (w/w) ratio using the Sylgard 184 Silicone Elastomer Kit (Dow Corning, USA).^[^ ^13^, ^36^ ^]^ It was subjected to vacuum desiccation for 30 min to remove bubbles from the mixture. The mixture was poured gently onto the mold and baked at 70 °C for 45 min. The microfluidics chip and coculture models were fabricated using a 2‐layer process.^[^ ^35^ ^]^

### Bacteria and Growth Conditions

The bacterial strains used in this study are listed in Table S1 (Supporting Information). Bacteria were cultivated in 2 mL Luria‐Bertani (LB) medium (Difco. Becton Dickinson and Company, USA) with the corresponding antibiotics at 37 °C and 200 rpm shaking overnight.^[^ ^37^ ^]^ The overnight bacterial culture was centrifuged at 10 000 *g* for 3 min, and the LB supernatant was removed. The cells were washed in 0.9% NaCl (w/v) saline and resuspended in RPMI antibiotics‐free (10% FBS) medium before being introduced into the tumor spheroids.

### Cell Culture and Cultivation

The human non‐small cell lung cancer cell line (A549) (CCL‐185, ATCC) was maintained in Roswell Park Memorial Institute (RPMI) medium (Gibco, UK) supplemented with 10% (v/v) fetal bovine serum (FBS; Gibco, USA) and 1% (v/v) penicillin–streptomycin (Gibco, USA) at 37 °C, 5% CO_2_ and 99% humidity.^[^ ^38^ ^]^ The cells were cultured in sterile T25 and T75 culture flasks (Thermofisher, USA). The medium was replaced every 48 h, and cells were passaged at 80% confluence. For seeding prior to the start of the experiment, A549 cells were diluted to a concentration of 4 × 10^5^ cells mL^−1^ and 1 × 10^5^ cells were seeded into each channel.

### Establishment of the Tumor‐Bacteria Coculture Model

The multiplicity of infection (MOI) used for initial coculture was 1 bacterium: 10^5^ tumor cells. The control group was seeded directly into the microchannels without introducing bacteria. The cocultures were then incubated at 37 °C, 5% CO_2_ and 99% humidity for 0, 1, 4, and 8 h. The media was replenished every 2 h to ensure minimal nutrient depletion.

### Chemical Treatment on a Tumor‐Tumor‐Associated Bacteria Coculture Model

For chemical treatment, the 2,2′‐bipyridine (DIPY) (10 × 10^−6^ m) (Sigma‐Aldrich, USA), FeCl_3_ (10 × 10^−6^ m), pyoverdine (50 × 10^−6^ m) (Sigma‐Aldrich, USA), or ferrostatin (10 × 10^−6^ m) (Abcam, USA) were added to the co‐cultures at 8 h.p.i for a further 8 h at 37 °C, 5% CO_2_ and 99% humidity.

For elimination of tumor‐tumor‐associated bacterial co‐culture model using the triple treatment combinational therapy, the cocultures were directly treated with 10 × 10^−6^ m thiostrepton (TST) (Abcam, USA), 8 µg mL^−1^ tobramycin (Tob) (Sigma‐Aldrich, USA) and 2.5 × 10^−6^ m doxorubicin (DOX) for a further 8 h at 37 °C, 5% CO_2,_ and 99% humidity.

### Imaging Coculture Model Using CLSM

To observe the co‐culture model, 1 × 10^−6^ m Cell Tracker Deep Red (Invitrogen, USA; Ex: 641 nm; Em: 662 nm) was added to the cells to stain all human cells. The 20 × 10^−6^ m propidium iodide (PI) (Invitrogen, USA; Ex: 535 nm; Em: 617 nm) on RPMI antibiotics‐free medium was used to stain dead tumor cells. Representative images of the cocultures were captured using the Leica TCS SP8 MP Multiphoton/Confocal Microscope (Germany) with a 63× oil objective to monitor brightfield, bacterial GFP, bacterial pyoverdine, PI, and Cell Tracker channels. ImageJ was used to process the images. Relative fluorescence levels were quantified using ImageJ with the equation of corrected total cell fluorescence (CTCF) = integrated density ‐ (area of selected cell x mean fluorescence background signals).

### Quantification of Intracellular Iron Concentration in Tumor

At 8 h.p.i, the spent culture medium was discarded, and the cells were washed with 1× PBS 3 times. The cell suspensions were transferred into centrifuge tubes, followed by centrifugation at 2000 rpm for 3 min. The bacteria‐contained supernatants were removed, and the cell pellets were resuspended in PBS. Only tumor cells were lysed by sonication at 40% amplitude for a total of 10 min (10 s ON/10 s OFF, stop for 3 min for cooldown) in the ice‐cold slurry by using an ultrasonication machine (bowl type, SFX 550, Branson, USA). The iron concentrations of tumor cells were measured via the Iron test kit (Sigma‐Aldrich, USA) according to the manufacturer's instructions. The treated samples were measured absorbance at OD595 nm via a microplate reader (Infinite M1000 Pro, Tecan, Switzerland) in a 96‐well plate (SPL, Korea).

### Colony‐Forming Unit (CFU) Assay

The 1x PBS was first used to wash the channels 3 times to remove unattached bacterial cells. The sterile ddH_2_O was added into channels to lyse the tumor cells and release tumor‐associated bacteria into the suspension. As previously described,^[^ ^39^ ^]^ the bacterial suspensions were serially diluted with 0.9% NaCl (w/v), then plated on LB agar plates at 37 °C for 16 h. After enumerating the colonies on the agar plates, the CFU mL^−1^ was calculated by the average number of colonies x dilution factor / total volume.

### Quantification of Reactive Oxygen Species (ROS) in Tumor Cells

The ROS levels in tumor cells were quantified through DCFDA/H2DCFDA—Cellular ROS assay kit (Abcam, USA) according to manufacturer's instructions.^[^ ^40^ ^]^ 20 × 10^−6^ m DCFDA was added to the microchannel for 45 min at 37 °C with 5% CO_2_. The fluorescence levels of the medium on microchannels were measured via a microplate reader (Tecan, Denmark; Ex: 485 nm; Em: 535 nm) in a 96‐well plate (SPL Korea).

### Quantification of c‐di‐GMP Level in Tumor‐Associated Bacteria

The 1x PBS was first used to wash the channels 3 times to remove unattached bacterial cells. The sterile ddH_2_O was added into channels to lyse the tumor cells and release tumor‐associated bacteria into the suspension. All suspensions were lysed through an ultrasonication machine (bowl type, SFX 550, Branson, USA) at 40% amplitude for 10 min (10 s ON/10s OFF, stop for 3 min for cooldown). The c‐di‐GMP level of all samples was quantified using the c‐di‐GMP ELISA kit (LMAI, Shanghai, China) according to the manufacturer's instructions.^[^ ^41^ ^]^ and the samples were measured at OD450 nm with the microplate reader (Infinite M1000 Pro, Tecan, Switzerland). The expression levels of c‐di‐GMP were normalized by total protein concentration.

### Quantification of Human Transferrin Receptor Expression

The 1x PBS was first used to wash the channels 3 times to remove unattached bacterial cells. The sterile ddH_2_O was then added into channels to lyse the tumor cells. The expression level of the human transferrin receptor in each sample was quantified using the Human Transferrin Receptor ELISA kit (Abcam, USA) according to manufacturer's instructions. The samples were measured at OD450 nm in the ELISA 96‐well plate using the microplate reader (Infinite M1000 Pro, Tecan, Switzerland). The levels of human transferrin receptors were normalized by total protein concentration by measuring absorbance at OD280mm via a Nanodrop detector (Thermofisher, USA).

### Statistical Analysis

Experiments were performed in triplicate. Averages and standard deviations were calculated using Microsoft Excel. The one‐way ANOVA and Student *t*‐test (paired) were calculated using Graphpad Prism, where appropriate. All data and figures are shown as the mean±s.d.

## Conflict of Interest

The authors declare no conflict of interest.

## Supporting information

Supporting Information

## Data Availability

The data that support the findings of this study are available from the corresponding author upon reasonable request.
