# Supplementary material for: Bacterial Iron Siderophore Drives Tumor Survival and Ferroptosis Resistance in a Biofilm‐Tumor Spheroid Coculture Model
Source: Adv Sci (Weinh). 2024 Aug 12;11(39):2404467. doi: 10.1002/advs.202404467 (PMC11496991; doi:10.1002/advs.202404467)
Supplement: Supplementary file 1 — Supporting Information [file ADVS-11-2404467-s001.docx]

**Supplementary Figures**


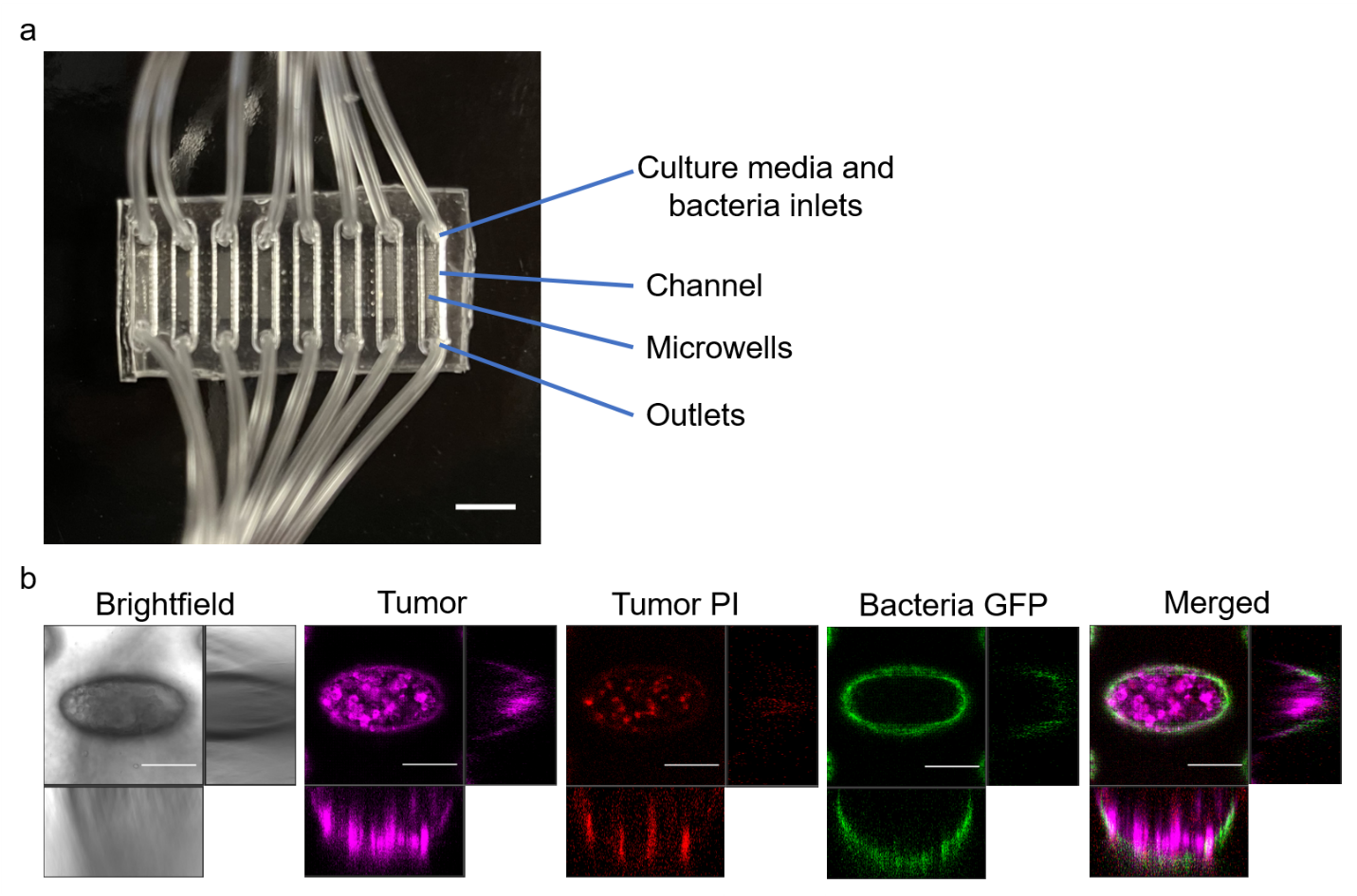


**Supplementary Figure 1. Cultivation of tumor spheroid-bacterial coculture in the microfluidics device.** (a) Photo of the microfluidics device. Scale bar: 1 cm. (b) Representative images of the tumor spheroid-bacterial coculture in a microwell. Scale bar: 100 µm.


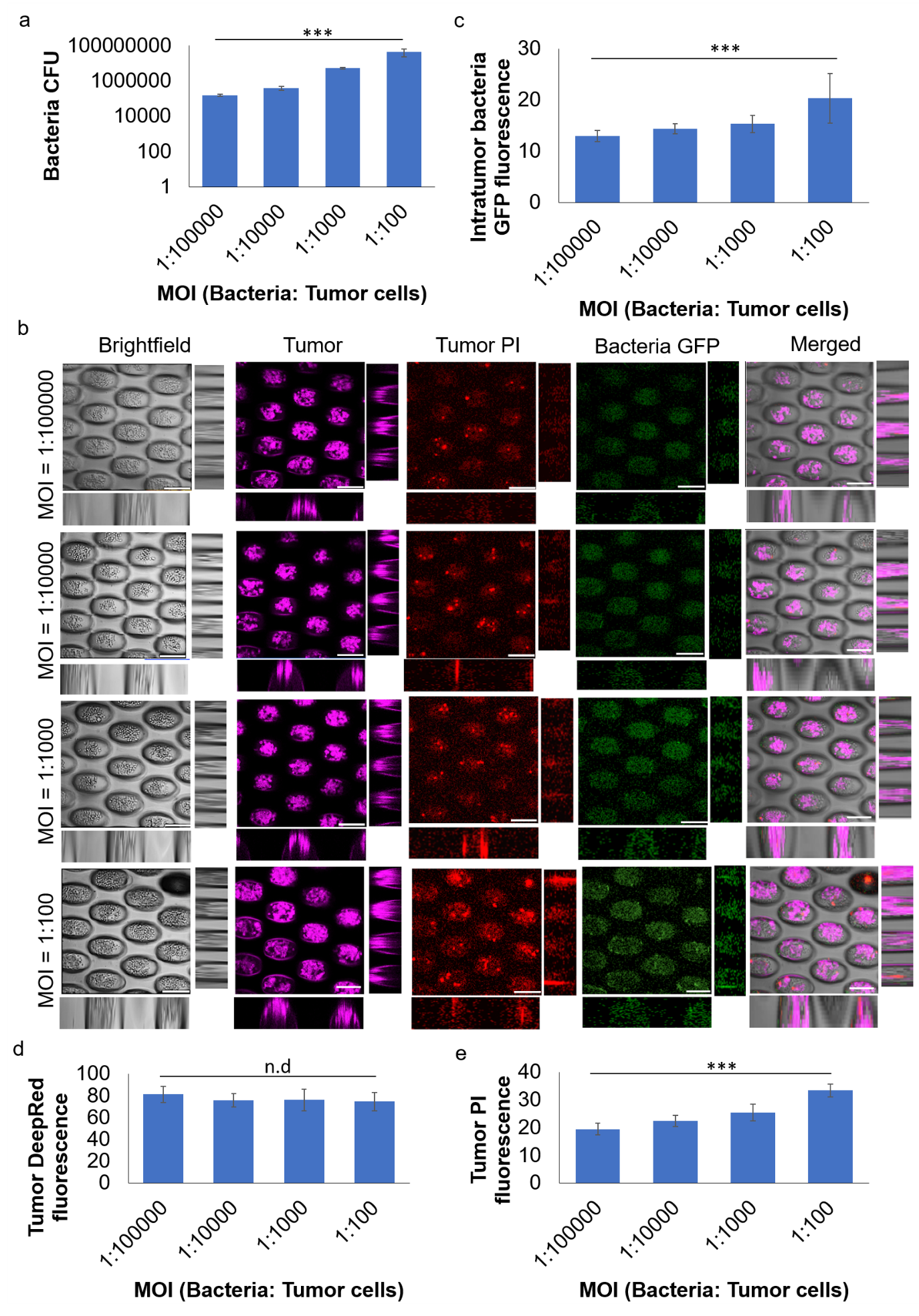


**Supplementary Figure 2. Different MOIs of tumor-bacterial coculture model.** (a) Bacterial CFU of intra-tumor *P. aeruginosa* in lung tumor spheroid at various MOIs. (b) Representative images of 3D-lung tumor spheroid-intratumor *P. aeruginosa* coculture model at various MOIs. Scale bar: 200 µm. (c) Relative GFP fluorescence levels of intra-tumor *P. aeruginosa* in lung tumor spheroid at various MOIs. (d) Relative Deep-Red fluorescence levels of lung tumor spheroids in tumor-bacterial coculture model at various MOIs. (e) Relative PI fluorescence levels of lung tumor spheroids in tumor-bacterial coculture model at various MOIs. The means and s.d. from triplicate experiments from 3 independent trials were shown. ****p* < 0.001. N.s: not significant.


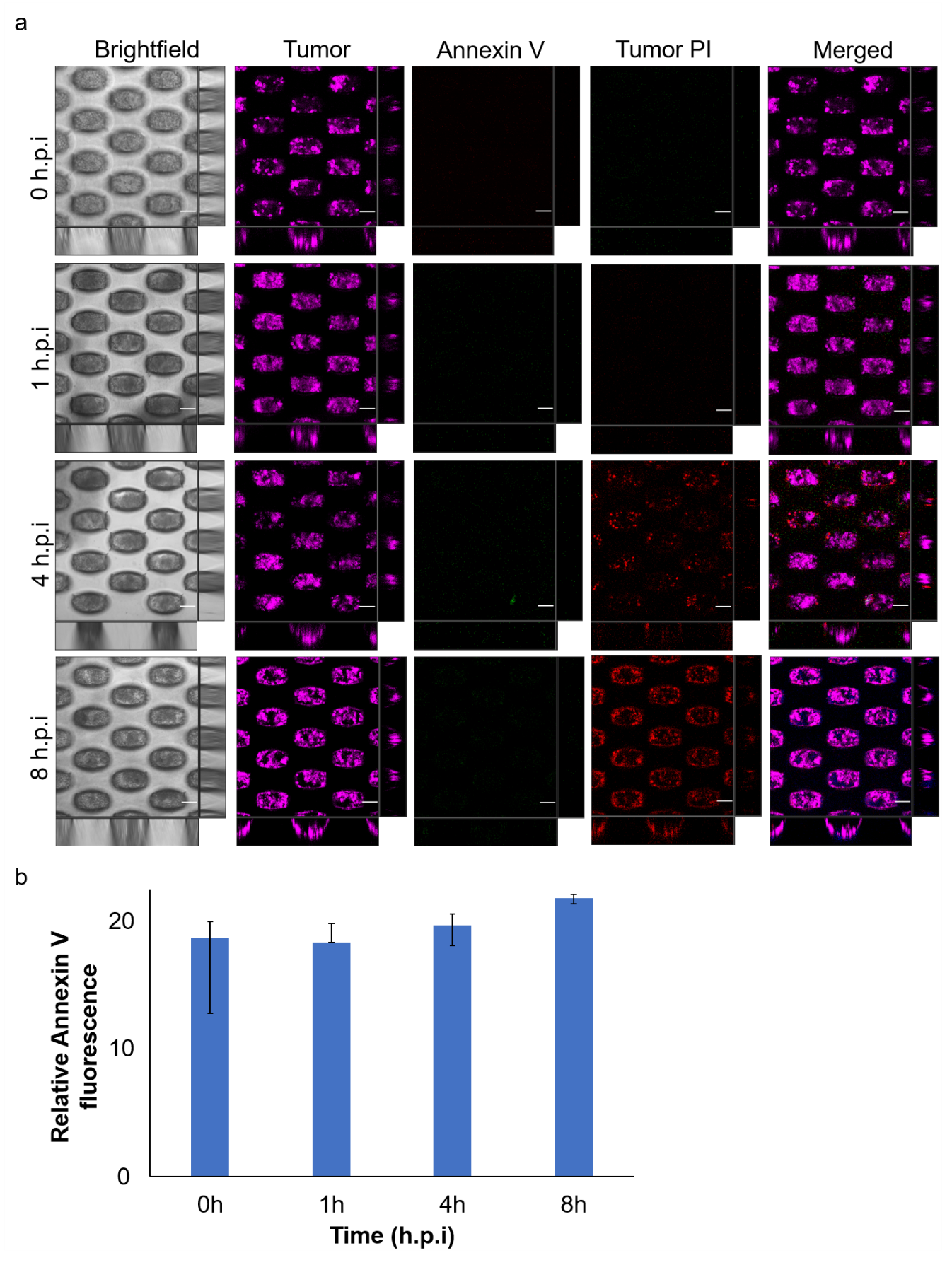
 **Supplementary Figure 3. Apoptosis was not detected in the 3D-lung tumor spheroid-intratumor *P. aeruginosa* coculture model.** (a) Representative images of 3D-lung tumor spheroid-intratumor *P. aeruginosa* coculture model with Annexin V apoptotic stain over time. Scale bar: 100 µm. (b) Relative Annexin V fluorescence levels of the lung tumor spheroids. The means and s.d. from triplicate experiments from 3 independent trials were shown.


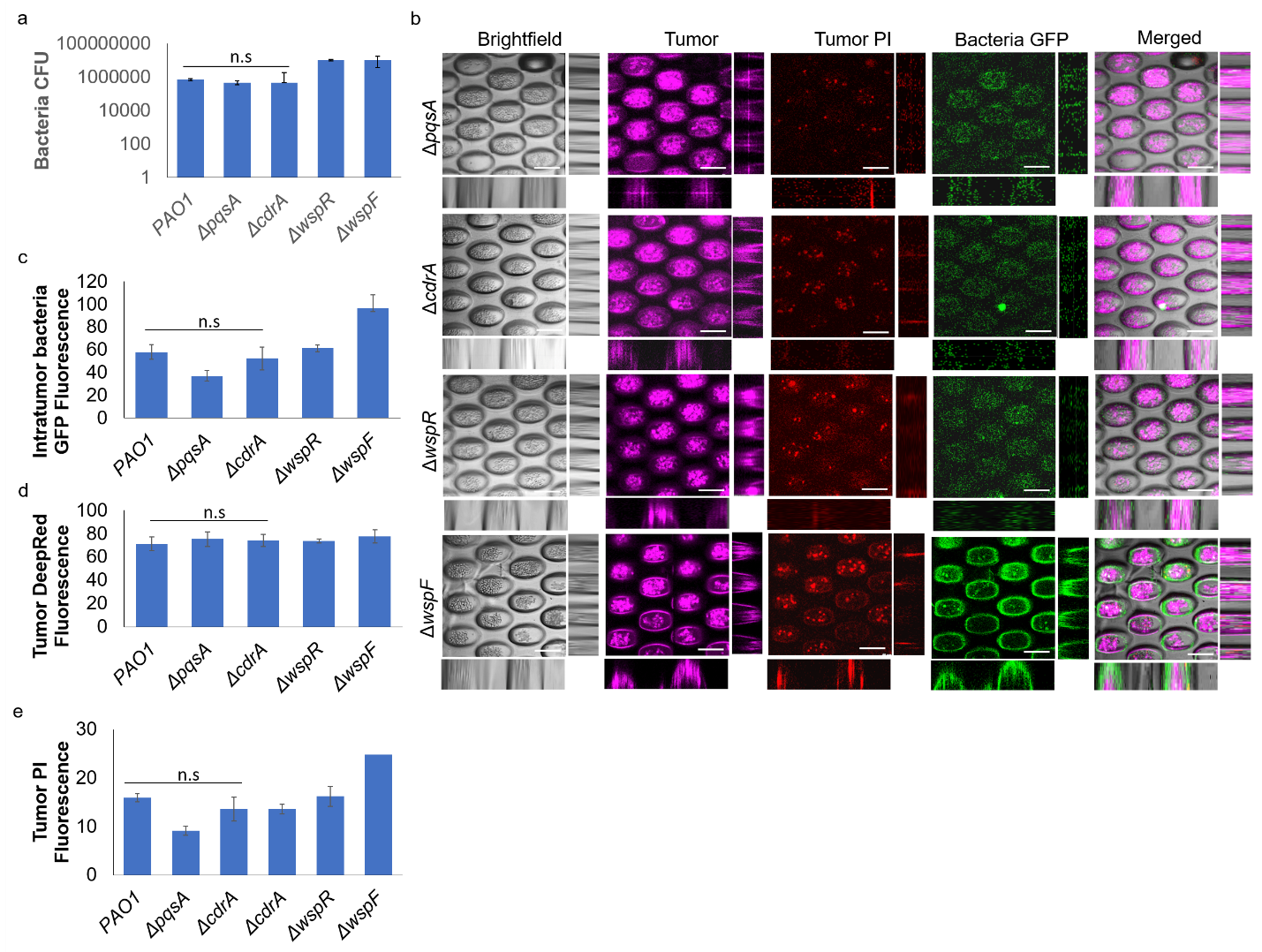
**Supplementary Figure 4. Screening of biofilm mutant library in tumor-biofilm coculture model.** (a) Bacterial CFU of intra-tumor *P. aeruginosa* in lung tumor spheroid. (b) Representative images of 3D-lung tumor spheroid-intratumor *P. aeruginosa* coculture model using different bacterial biofilm mutants. Scale bar: 200 µm. (c) Relative GFP fluorescence levels of intra-tumor *P. aeruginosa* in lung tumor spheroid using different bacterial biofilm mutants. (d) Relative Deep-Red fluorescence levels of lung tumor spheroids in tumor-bacterial coculture model using different bacterial biofilm mutants. (e) Relative PI fluorescence levels of lung tumor spheroids in tumor-bacterial coculture model using different bacterial biofilm mutants. The means and s.d. from triplicate experiments from 3 independent trials were shown. N.s: not significant.


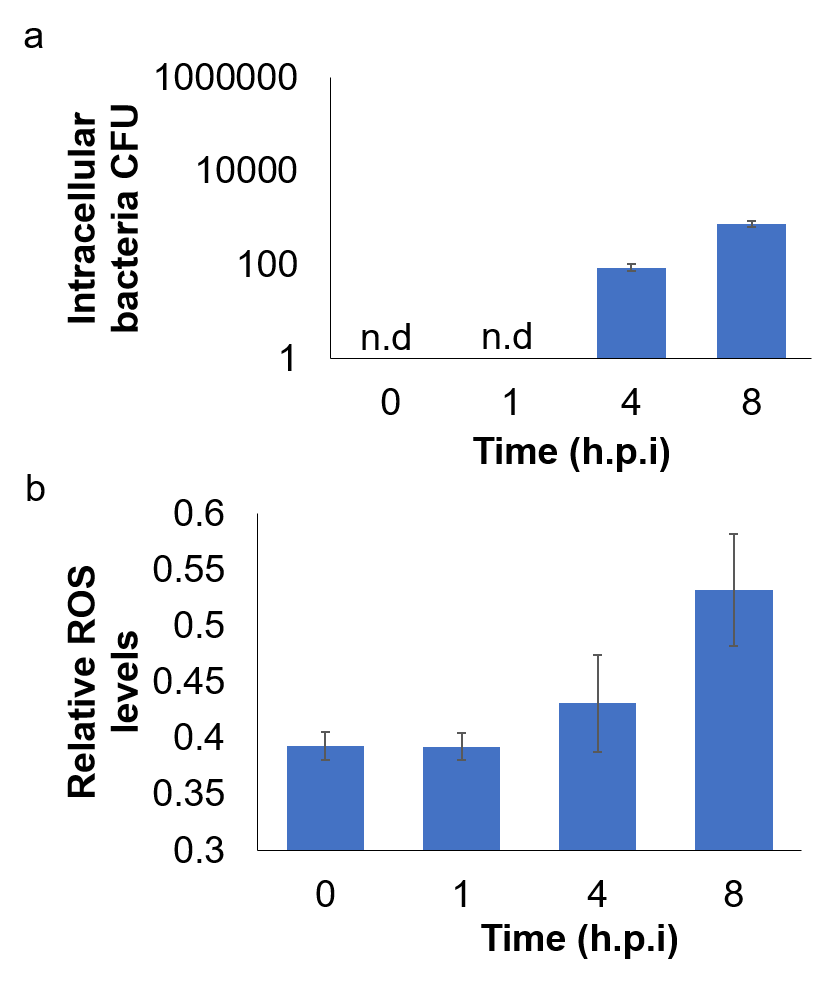


**Supplementary Figure 5. Minimal presence of intracellular bacteria within the tumor cells.** (a) Population number of intracellular *P. aeruginosa* within lung tumor cells. (b) Relative ROS levels within tumor cells containing intracellular bacteria. The means and s.d. from triplicate experiments from 3 independent trials were shown. N.d: not detected.


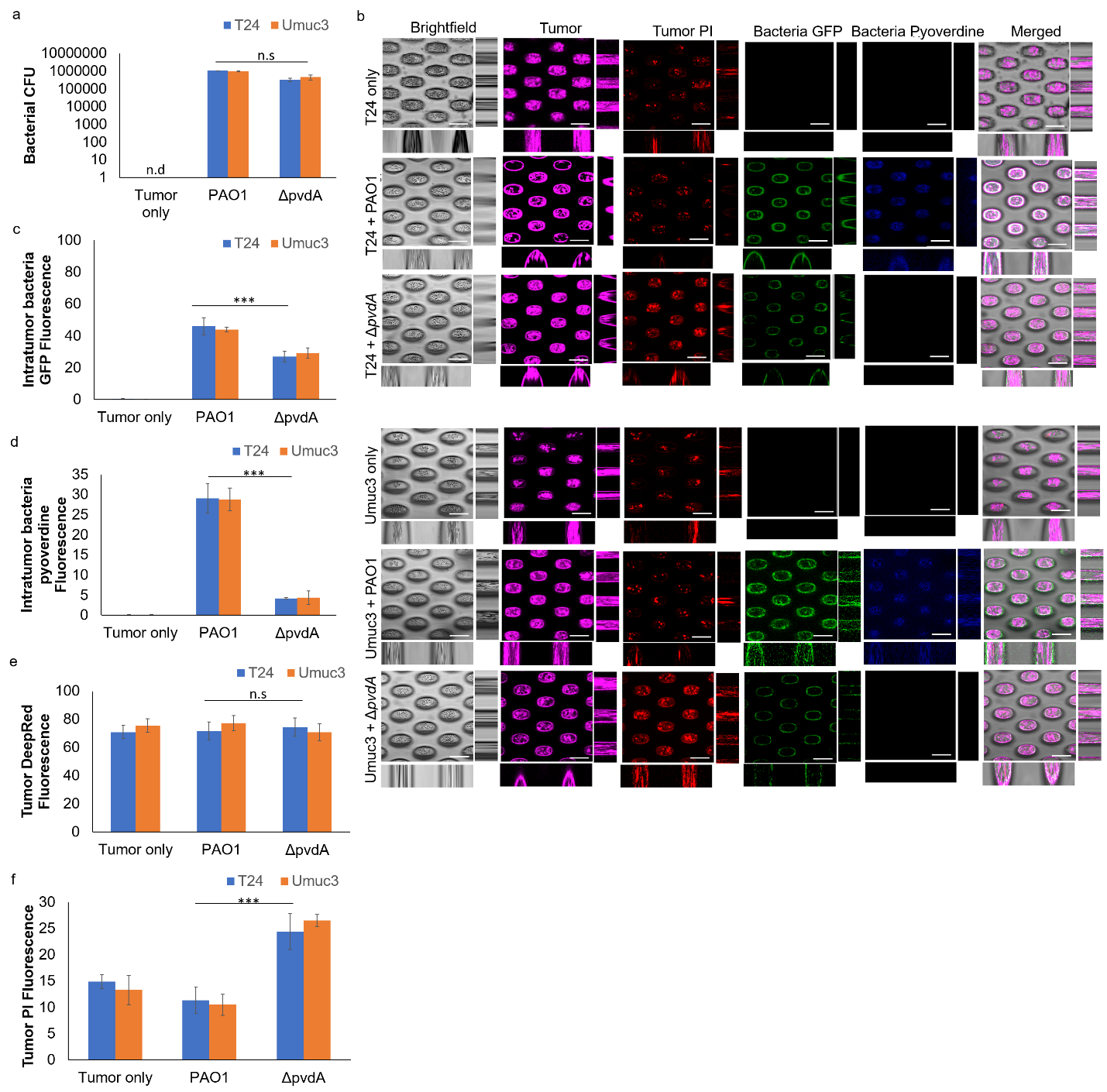


**Supplementary Figure 6. *P. aeruginosa* is important in ferroptosis suppression in different cancer cell lines (T24 and Umuc3).** (a) Bacterial CFU of intra-tumor *P. aeruginosa* in tumor spheroids from different tumor cell lines. (b) Representative images of 3D-tumor spheroid-intratumor *P. aeruginosa* coculture model using different tumor cell lines. Scale bar: 200 µm. (c) Relative GFP fluorescence levels of intra-tumor *P. aeruginosa* in tumor spheroid using different tumor cell lines. (d) Relative pyoverdine fluorescence levels of intra-tumor *P. aeruginosa* in tumor spheroid using different tumor cell lines. (e) Relative Deep-Red fluorescence levels of tumor spheroids in tumor-bacterial coculture model using different tumor cell lines. (f) Relative PI fluorescence levels of tumor spheroids in tumor-bacterial coculture model using different tumor cell lines. The means and s.d. from triplicate experiments from 3 independent trials were shown. ****p* < 0.001. N.s: not significant.


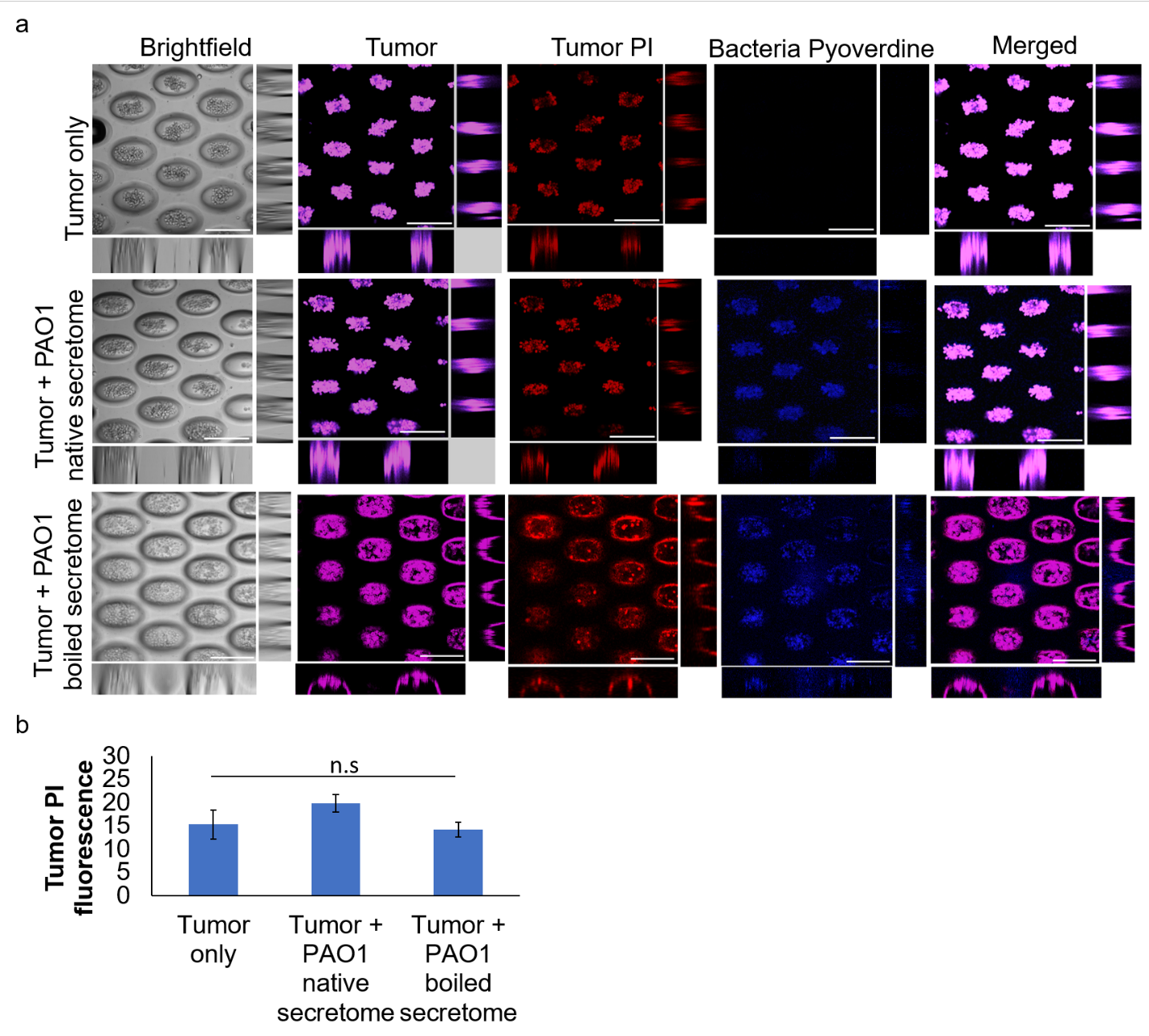


**Supplementary Figure 7. *P. aeruginosa* native secretome is involved in ferroptosis suppression of tumors.** (a) Representative images of 3D-lung tumor spheroids treated with native and boiled *P. aeruginosa* secretomes. Scale bar: 200 µm.

(b) Relative PI fluorescence levels of lung tumor spheroids treated with native and boiled *P. aeruginosa* secretomes. The means and s.d. from triplicate experiments from 3 independent trials were shown. N.s: not significant.


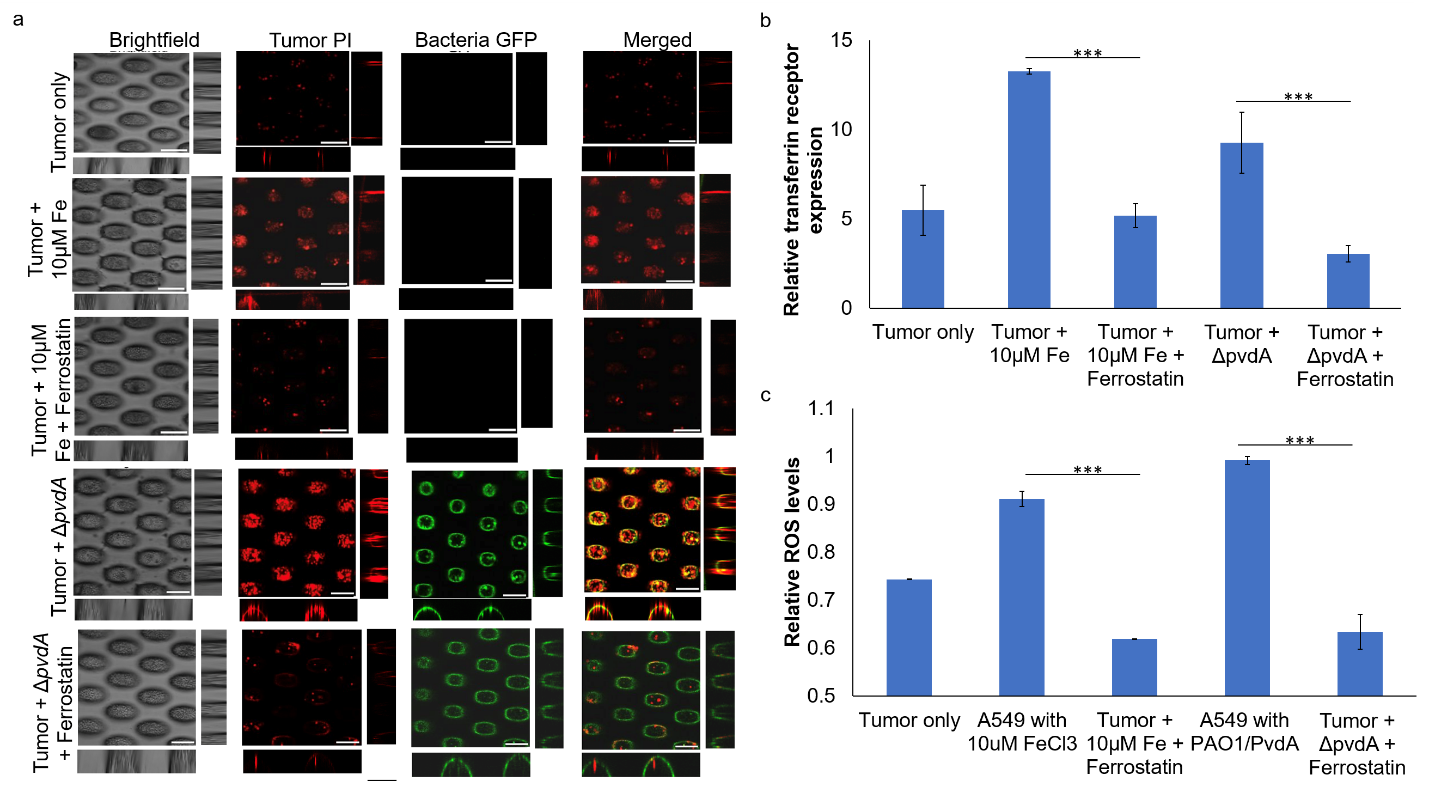


**Supplementary Figure 8.** **Ferrostatin could suppress ferroptosis in tumor cells treated with exogenous iron or cultivated with Δ*pvdA* mutant.** (a) Representative images of ferrostatin-treated-lung tumor spheroids after exposure to exogenous iron or Δ*pvdA* mutant. Scale bar: 200 µm. (b) Relative transferrin receptor expression by ferrostatin-treated tumor cells after exposure to exogenous iron or Δ*pvdA* mutant. (c) Relative ROS levels within ferrostatin-treated tumor cells after exposure to exogenous iron or Δ*pvdA* mutant. The means and s.d. from triplicate experiments from 3 independent trials were shown. ****p* < 0.001.


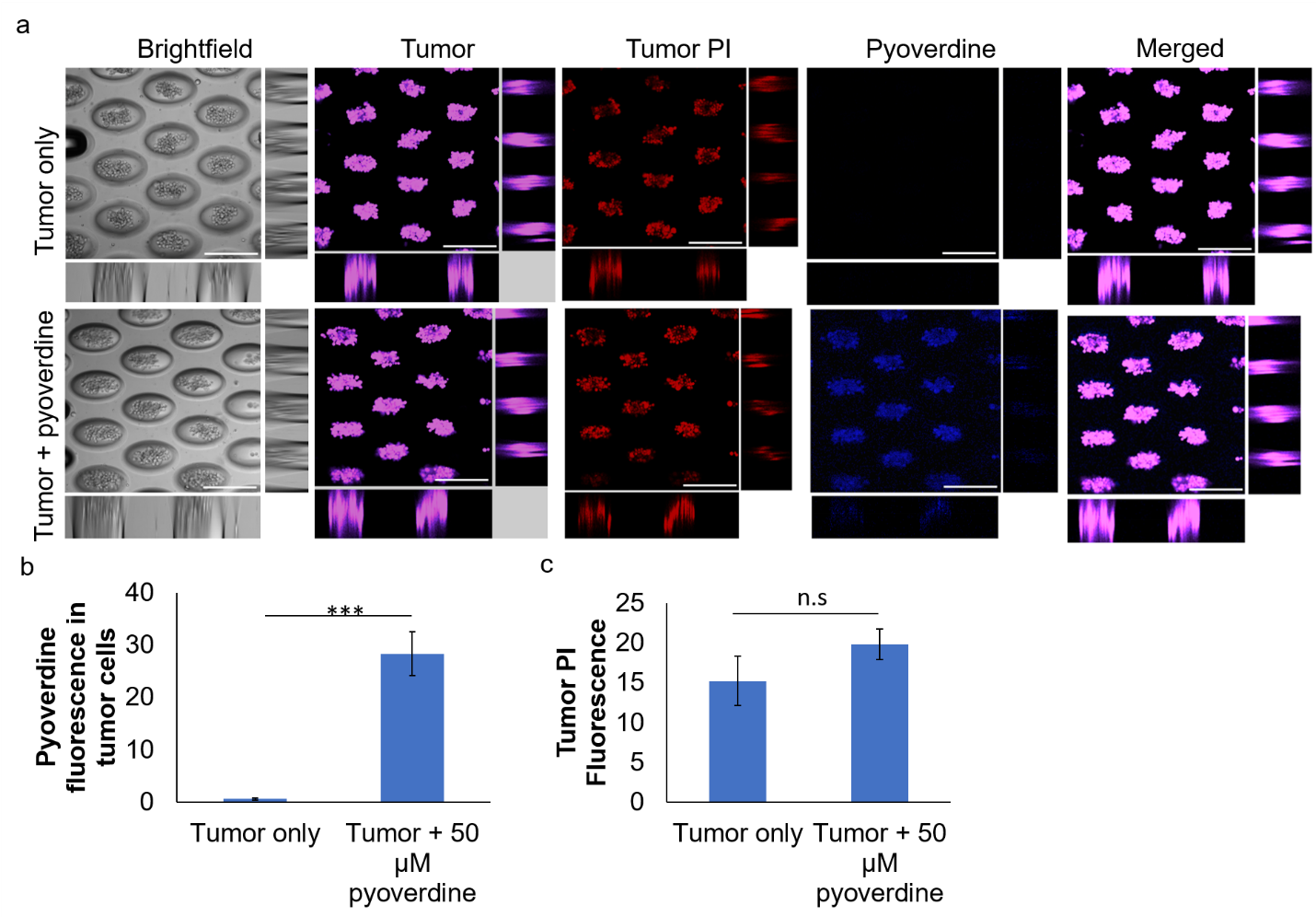


**Supplementary Figure 9. Pyoverdine could enter into tumor cells.** (a) Representative images of lung tumor spheroids treated with exogenous pyoverdine. Scale bar: 200 µm. (b) Relative pyoverdine fluorescence levels in lung tumor spheroids treated with exogenous pyoverdine. (c) Minimal cytotoxicity of pyoverdine to tumor cells. The means and s.d. from triplicate experiments from 3 independent trials were shown. ****p* < 0.001. N.s: not significant.


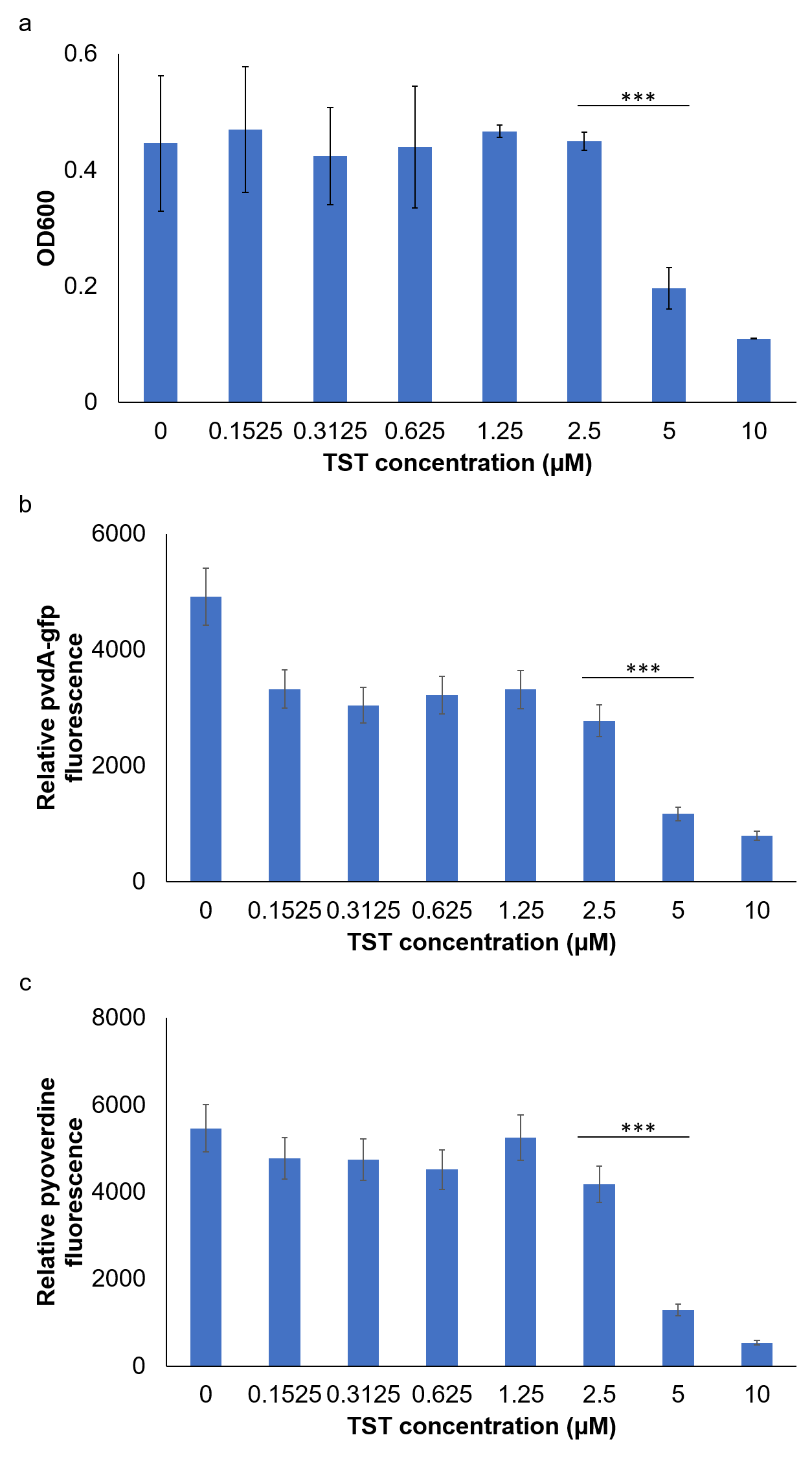


**Supplementary Figure 10. Thiostrepton (TST) inhibits pyoverdine production by *P. aeruginosa*, but does not kill bacteria.** (a) Relative GFP expression of PAO1/p*_pvdA_*-*gfp* after TST treatment. (b) Relative pyoverdine expression of PAO1/p*_pvdA_*-*gfp* after TST treatment. (c) Bacterial numbers after TST treatment using the CFU assay. The means and s.d. from triplicate experiments from 3 independent trials were shown. ****p* < 0.001.


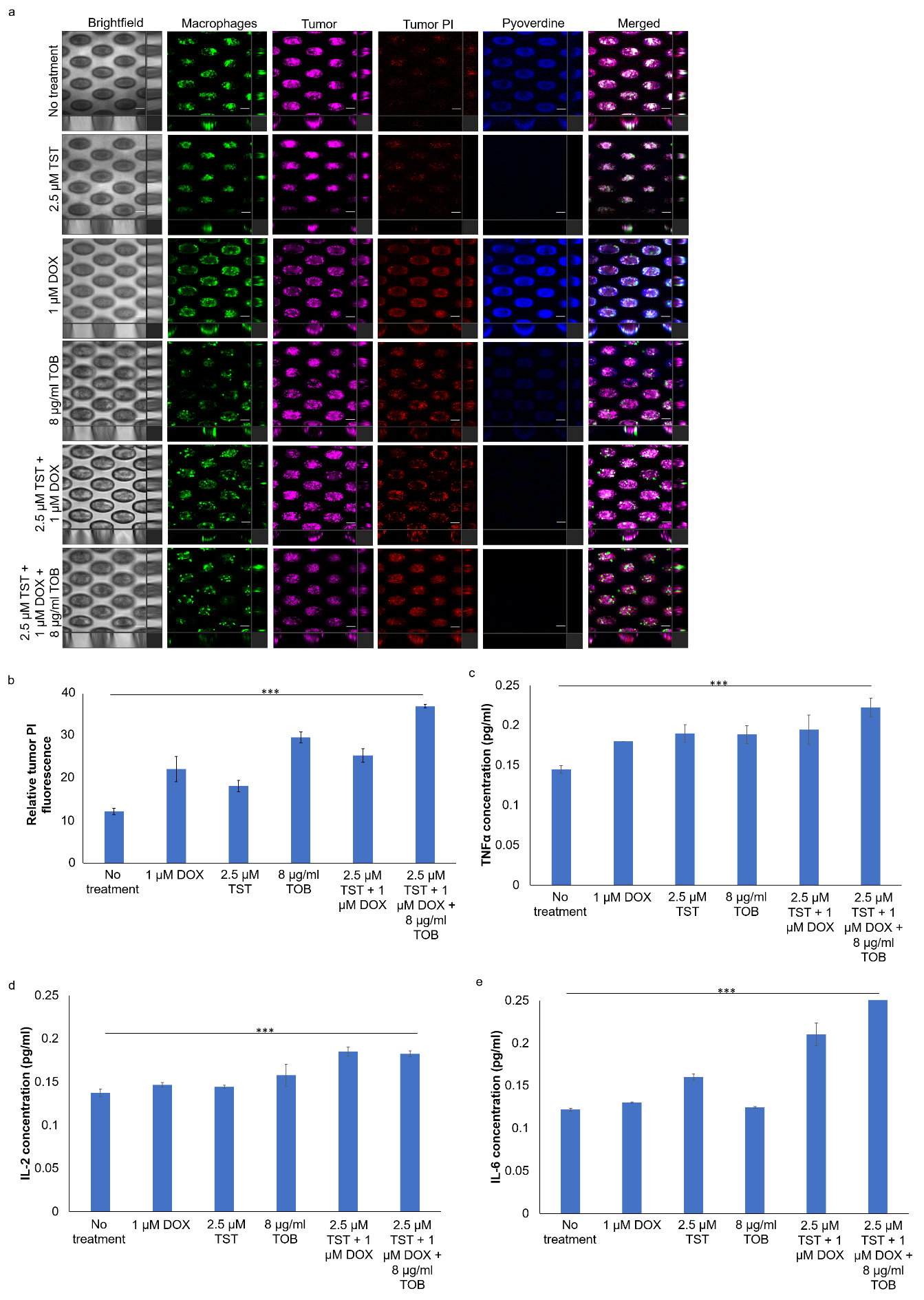


**Supplementary Figure 11. Triple treatment of Thiostrepton (TST), tobramycin (TOB) and doxorubicin (DOX) can eliminate tumor-bacterial co-culture and promote cytokine activation by macrophages.** (a) Representative images of tumor-macrophage-bacterial co-culture. Scale bar: 100 µm. (b) Relative PI fluorescence levels of lung tumor spheroids treated with various compounds. Concentrations of macrophage cytokines (c) TNF-α, (d) IL-2 and (e) IL-6 after compound treatment. The means and s.d. from triplicate experiments from 3 independent trials were shown. ****p* < 0.001.

**Supplementary Table 1.** Bacterial strains used in this study.

| Strain / plasmid  (*P. aeruginosa*) | Description | Source |
| --- | --- | --- |
| *PAO1* | Wild-type prototypic nonmucoid strain | ^1^ |
| *ΔpvdA* | Allelic exchange constructed mutant *PAO1* without *pvdA* | ^2^ |
| *ΔpvdA*/*pvdA* Com | Tc^r^, Allelic exchange constructed mutant *PAO1* without *pvdA*, with *pvdA* complementary plasmid (pME6031) | ^2^ |
| PAO1/p*_lac_*-*gfp* | Gm^r^, PAO1 containing the Tn7-transposon (p*_lac_-gfp*), with constitutively expressed GFP | ^3^ |
| PAO1*/* p*_cdrA_*-*gfp* | Gm^r^, PAO1 with p*_cdrA_*-*gfp* transcriptional fluorescent fusion biosensor | ^4^ |
| PAO1/p*_pvdA_*-*gfp* | Tc^r^, PAO1 with p*_pvdA_*-*gfp* transcriptional fluorescent fusion biosensor | ^5^ |
| Δ*pvdA/*p*_pvdA_*-*gfp* | Tc^r^, *ΔpvdA* with p*_pvdA_*-*gfp* transcriptional fluorescent fusion biosensor | ^5^ |
| Δ*pvdA/*p*_lac_*-*gfp* | Gm^r^, *pvdA* knockout of PAO1 constructed by  allelic exchange, carrying the p*_lac_*-*gfp* reporter | ^5^ |
| Δ*pelA/*p*_lac_*-*gfp* | Gm^r^; *pelA* knockout of PAO1 constructed by  allelic exchange, carrying the p*_lac_*-*gfp* reporter | ^4^ |
| Δ*pslBCD/*p*_lac_*-*gfp* | Gm^r^; *pslBCD* knockout of PAO1 constructed by  allelic exchange, carrying the p*_lac_*-*gfp* reporter | ^4^ |
| Δ*pelA*Δ*pslBCD/*p*_lac_*-*gfp* | Gm^r^; *pelA* and *pslBCD* knockout of PAO1 constructed by  allelic exchange, carrying the p*_lac_*-*gfp* reporter | ^4^ |
| Δ*cdrA/*p*_lac_*-*gfp* | Gm^r^; *cdrA* knockout of PAO1 constructed by  allelic exchange, carrying the p*_lac_*-*gfp* reporter | ^4^ |
| Δ*pqsA/*p*_lac_*-*gfp* | Gm^r^; *pqsA* knockout of PAO1 constructed by  allelic exchange, carrying the p*_lac_*-*gfp* reporter | ^4^ |
| Δ*wspR/*p*_lac_*-*gfp* | Gm^r^; *wspR* knockout of PAO1 constructed by  allelic exchange, carrying the p*_lac_*-*gfp* reporter | ^4^ |
| Δ*wspF/*p*_lac_*-*gfp* | Gm^r^; *wspF* knockout of PAO1 constructed by  allelic exchange, carrying the p*_lac_*-*gfp* reporter | ^4^ |

**References**

1. Holloway BW, Morgan AF. Genome Organization in *Pseudomonas*. *Annual Review of Microbiology* **40**, 79-105 (1986).

2. Visca P, Ciervo A, Orsi N. Cloning and nucleotide sequence of the pvdA gene encoding the pyoverdin biosynthetic enzyme L-ornithine N5-oxygenase in *Pseudomonas aeruginosa*. *Journal of Bacteriology* **176**, 1128 (1994).

3. Lambertsen L, Sternberg C, Molin S. Mini-Tn7 transposons for site-specific tagging of bacteria with fluorescent proteins. *Environmental Microbiology* **6**, 726-732 (2004).

4. Ma Y, Deng Y, Hua H, Khoo BL, Chua SL. Distinct bacterial population dynamics and disease dissemination after biofilm dispersal and disassembly. *The ISME Journal*, (2023).

5. Chen Y*, et al.* Multiple diguanylate cyclase-coordinated regulation of pyoverdine synthesis in *Pseudomonas aeruginosa*. *Environmental microbiology reports* **7**, 498-507 (2015).
